# A Multi-Skilled Mathematical Model of Bacterial Attachment in Initiation of Biofilms

**DOI:** 10.3390/microorganisms10040686

**Published:** 2022-03-23

**Authors:** Kanchana Chathoth, Louis Fostier, Bénédicte Martin, Christine Baysse, Fabrice Mahé

**Affiliations:** 1 CIMIAD, NUMECAN INSERM U1241, Université de Rennes 1, F-35043 Rennes, France; kanchanachathoth@gmail.com (K.C.); benedicte.martin@univ-rennes1.fr (B.M.); christine.baysse@univ-rennes1.fr (C.B.); 2 IRMAR, CNRS UMR 6625, Université de Rennes, F-35000 Rennes, France; louis.fostier@etudiant.univ-rennes1.fr

**Keywords:** biofilm, bacterial attachment, mathematical model, *Porphyromonas gingivalis*, *Streptococcus gordonii*, *Treponema denticola*, iron

## Abstract

The initial step of biofilm formation is bacteria attachment to biotic or abiotic surfaces and other bacteria through intra or interspecies interactions. Adhesion can be influenced by physicochemical conditions of the environment, such as iron. There is no available mathematical model of bacterial attachment giving realistic initiation rather than random adhesion. We describe a simple stochastic attachment model, from the simplest case in two dimensions with one bacterial species attaching on a homogeneous flat surface to more complex situations, with either several bacterial species, inhomogeneous or non-flat surfaces, or in three dimensions. The model depends on attachment probabilities (on the surface, laterally, or vertically on bacteria). Effects of each of these parameters were analyzed. This mathematical model is then applied to experimental oral microcolonies of *Porphyromonas gingivalis*, *Streptococcus gordonii*, and *Treponema denticola*, either as mono-, two, or three species, under different iron concentrations. The model allows to characterize the adhesion of three bacterial species and explore the effect of iron on attachment. This model appears as a powerful tool for initial attachment analysis of bacterial species. It will enable further modeling of biofilm formation in later steps with biofilm initialization more relevant to real-life subgingival biofilms.

## 1. Introduction

Biofilms are characterized by a community of microorganisms attached to a surface or located at a liquid/air interface and are generally covered by an extracellular matrix of exopolysaccharides (EPS), proteins, DNA, and membrane vesicles. Biofilms are the preferred communal lifestyle of microorganisms [1]. These biofilms affect both the industrial sector (bioerosion, biofouling), the public health sector (infections, contamination of medical materials), and the ecological sector (complex ecosystems, pollution control) [1,2,3]. The formation of a biofilm goes through several stages, starting with the attachment of planktonic cells alone or co-aggregated, the formation of microcolonies, the expansion of these microcolonies, and the maturation of the biofilm by the production of extracellular matrix. The last phase involves a detachment of sessile cells which are returning to the planktonic state and/or a detachment of sessile cells still embedded in the matrix.

Bacterial attachment to biotic or abiotic surfaces is the initial step of biofilm formation. Biofilm initiation by adhesion depends on both bacterial cell characteristics (electrical charge, cell surface components) and surface characteristics (charge, hydrophilicity, roughness) [4,5]. Adhesion is promoted by both fluid movement and/or cells motility towards the substratum, followed by electrostatic forces, hydrophobic bonds, and/or hydrogen bonds between the cells and the surface. In nature, multi-species biofilms are prevailing, and it is often observed that some species favor or inhibit other species for attachment. Each species has its own attachment rate on a specified surface, which is due to the presence of adhesin(s) at the cell surface and the overall electrical surface charge at a given pH value. The attachment process will affect the whole biofilm growth of a multi-species biofilm.

Multi-bacterial biofilms are complex physical and microbiological structures, in which many biological processes interact. The analysis of such interactions may be challenging. Mathematical modeling of biological processes is intended to gain insight into fundamental mechanisms by translation of conceptual hypothesis into equations that are as simple as possible. Because of their flexibility, mathematical models can be powerful tools to integrate a large amount of data corresponding to various biological processes and, by isolating one process from another, to evaluate their relative contribution in the biofilm growth.

Mathematical biofilms models are mainly focused on the growth phase of the process. Various studies have focused on the mathematical modeling of biofilm growth, taking into account different parameters such as biomass, bacterial composition, and/or spatial data. Two- or three-dimensional methods give access to the biofilm structure and can be classified into three types: continuous mechanical models [6,7], discrete models [8], and cellular automata [9,10,11,12]. In these models, biofilms are often initialized randomly on the surface, thus not considering the hierarchy or ability of each individual species to attach to the surface.

Adhesion of bacterial cells to the surface has been characterized by different physics and mathematics tools. In [13], the authors used thermodynamics to interpret experimental data of *Escherichia coli* adhesion according to surface parameters. Mathematical ordinal regression was also performed to evaluate the probability of adhesion of *Salmonella* species according to environmental conditions, such as pH, ionic concentrations, and temperature [14]. However, these studies do not include any spatial view of adhesion. The transition between planktonic and sessile status of bacteria was the subject of recent work [15] using modeling software (MAUDE), but still without any space visualization. Some complex mathematical models also deal with the influence of bacterial elastic properties and shape, and the topography of the surface on bacterial adhesion [16]. In most published studies, only bacterial adhesion to the surface was considered [17]. Recently, the authors of [18] investigated the probability of bacteria adhering to each other and linked some specific bacterial properties, such as surface sensitivity and EPS production, to the ability of bacteria to attach either to nearby (spatial neighbors) or progeny (temporal neighbors) cells.

To the best of our knowledge, no mathematical model of biofilm describes the initial adhesion of different bacterial species in a spatial model. Only authors of [19] published, in 2012, a comprehensive model of mono-species biofilm growth using a cellular automata model that includes adhesion in the process. In this model, the parameters taken into account to determine the probability of adhesion were the hydrodynamic properties of the fluid and the roughness of the surface.

A realistic mathematical model of adhesion, based on experimental data, is therefore lacking to fully reproduce a biofilm model from the initial step to its growth and maturation phases. The attachment process will affect the whole biofilm growth of a multi-species biofilm and must be included in the mathematical model.

In this paper, we present a simple stochastic model giving realistic initial biofilms described by their mean thickness, roughness, and biovolume. We first describe the algorithm in the simplest case of two dimensions with one bacterial species attaching on a homogeneous flat surface. The model is then extended to several bacterial species, and/or on inhomogeneous and non-flat surfaces, and/or in three dimensions. The model is dependent on three different parameters: probability of attachment on the surface, laterally on a bacterium, vertically on a bacterium. We analyze the effects of each of these parameters and give indications for the use of the method. We present various initial biofilms that can be obtained and discuss the limits of the model.

To show the relevance of the mathematical model to health issues, it is then applied to experimental oral biofilm initiation of three different species: *Porphyromonas gingivalis*, *Streptococcus gordonii*, and *Treponema denticola*. These species are involved in periodontal biofilms that can lead to teeth/bone loss.

A colonization hierarchy is established from the beginning of oral biofilm growth in oral biofilms, which starts with primary colonizers (*Streptococcus*), then secondary colonizers such as *Fusobacterium*, and eventually ends with the incorporation of anaerobic Gram-negative pathogens, responsible for periodontal diseases such as *P. gingivalis* and *T. denticola* [20,21,22,23,24]. The attachment and development of pathogens in the oral biofilm is therefore dependent on the attachment of primary and secondary colonizers.

Iron has recently been identified as an element capable of modifying the composition and virulence of oral biofilm and thus the severity of periodontal disease [25,26,27]. It cannot be excluded that iron influences the attachment of bacteria and therefore the initiation of biofilm. To assess the effect of iron on the initial stage of biofilm formation, the attachment model has been tested and experiments have been performed with different species and different iron concentrations.

## 2. Materials and Methods

### 2.1. Mathematical Model

Firstly, we present the attachment algorithm in the simplest case of only one species of bacterium attaching in a flat homogeneous surface with a two-dimension approximation. Then, we extend the model to several species of bacteria, inhomogeneous or non-flat surface, and three dimensions.

#### 2.1.1. Attachment Algorithm

**Figure 1:**
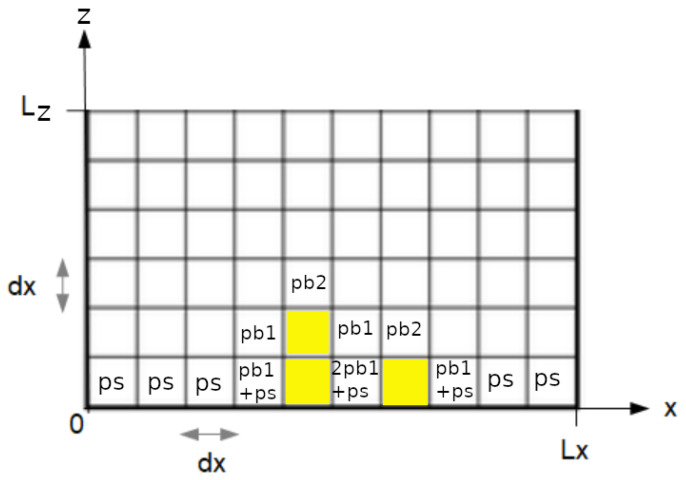
Attachment domain $\Omega =\left[0,L_{x}\right]\times \left[0,L_{z}\right]$, grid of the stochastic algorithm with, in yellow, the elements with attached bacteria and non-zero values of attachment probability of matrix Mpr.

The attachment of the bacteria is modeled by a stochastic process using a 2D lattice representing the domain of attachment $\Omega =\left[0,L_{x}\right]\times \left[0,L_{z}\right]$. $L_{z}$ is the height of the domain and $L_{x}$ is the width (see Figure 1). Each element of the lattice can contain one bacterial cell or an amount of bacteria depending on the size dx of the square element. This discretization of the domain is adapted to use a growth model based on cellular automata after the attachment phase [9,10]. The grid has $N_{x}=\frac{L_{x}}{dx}$ columns and $N_{z}=\frac{L_{z}}{dx}$ rows.

Each element of the grid is numbered by its position (i,j) in row *i* and column *j*. We define the matrix *b* such that b(i,j)=1 if there is bacteria in the element (i,j) and b(i,j)=0 if not. The process is dependent on three probabilities, ps, pb1, and pb2, for which the value is between 0 and 0.25:

- ps: the attachment probability on the surface.
- pb1: the probability of horizontal attachment on the side of an element occupied by bacteria.
- pb2: the probability of vertical attachment below or on the top of an element occupied by bacteria.

The attachment probability Mpr(i,j) for the element (i,j) is dependent on the four adjacent elements: below (i−1,j), top (i+1,j), left (i,j−1), and right (i,j+1). Mpr(i,j)=0 if there are no bacteria in the adjacent elements or if there are bacteria in (i,j). Each occupied adjacent element gives an additive contribution to Mpr(i,j): on the surface or substratum (i=1) there is a first contribution ps, pb1 is added for each occupied side element, and pb2 is added for each occupied element on the top and the bottom. See Figure 1 for an example of this attachment probability matrix. The probability of attachment increases with the number of occupied adjacent elements. To avoid edge effects, a periodic boundary condition is applied on the lateral boundaries (the element (i,1) is adjacent to element $\left(i,N_{x}\right)$).

The principle of the algorithm is simple: a possible attachment element (i,j) (with Mpr(i,j)≠0) is randomly selected and a Bernouilly test dependent on the value Mpr(i,j) is performed. If the test succeeds, then b(i,j)=1. The process is repeated until the desired number of occupied cells in the grid $Nb_{cell}$ is obtained (See Algorithm 1).
**Algorithm 1:** Attachment algorithm
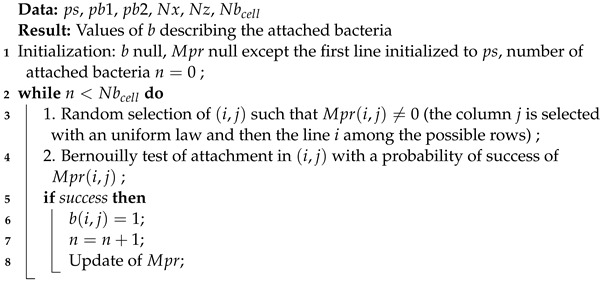

**Remark** **1.** *From this basic algorithm, different options can be proposed.*

*(i)*  *To save computing time, the place of the first bacterium attached to the surface can be chosen randomly without random Bernouilly test. In the same way, to force the number of microcolonies, several places can be initialized with bacteria on the surface.*
*(ii)*  *Instead of random selection of the row, it is possible to choose the first available place from the bottom in the selected column. This choice leads to more compact microcolonies without holes.*
*(iii)*  *Instead of running the process until a given number of bacteria are attached, a number (depending on the concentration of the medium) of attachment tests by minute can be chosen. Then, the number of attached bacteria depends on the time of the initialization process.*

#### 2.1.2. Model Extensions

This basic model can be easily extended to study more complex and general cases. Five extensions have been explored.

- *Several species of bacteria*. To simulate the attachment of *k* species of bacteria, more initial data are needed: the required number $Nb_{cell}$ of attached bacteria, the proportion prop(s) of the species *s*, the symmetrical matrix pb of size k×k×2 giving the probabilities of inter-bacterial attachment (pb(s,r,1) is the horizontal attachment probability of species *s* on species *r*, pb(s,r,2) is for the vertical attachment), and ps(s) the attachment probability on the substratum for the species *s*. There is an attachment probability matrix Mpr by species and b(i,j) can take on integer values from 0 to *k*. However, the algorithm is almost the same: the random choice of the species (with the constraint of respecting the given proportions) is added at the beginning of each iteration.
- *3D model of attachment*. The domain is a 3D straight block of size $L_{x}\times L_{y}\times L_{z}$ and the size of the matrices are adapted: *b* with size $N_{x}\times N_{y}\times N_{z}$, Mpr with size $N_{x}\times N_{y}\times N_{z}\times k$ if *k* species are present. In the algorithm, the choice of the column *j* is made in a 2D grid instead of a discretized line, the periodic conditions are applied on the four side boundaries, and the update of the attachment probability matrix Mpr is a bit more complex because each cube of the mesh has six adjacent elements.
- *Non-homogeneous surface*. If the attachment surface is made with different materials, it is only necessary to define a value of ps by material and adapt the initialization of matrix Mpr accordingly.
- *Non-flat surface*. The rectangular domain (or the block) is defined as previously but *b* and Mpr are initialized to indicate the position of the surface: b(i,j)=−1 and Mpr(i,j)=0 if the element (i,j) is filled with the material of the substratum.
- *Non-constant parameters of attachment*. Specific shapes can be obtained by varying the value of parameters in time or depending on the number of attached bacteria in the process. For instance, a tall mushroom shape is obtained with a very low horizontal attachment probability replaced by a high value after half of the attachment process.

### 2.2. Experimental Initial Bacteria Microcolonies

#### 2.2.1. Bacteria and Media

*Streptococcus gordonii* Challis DL1 [28], *Porphyromonas gingivalis* TDC60 [29], and *Treponema denticola* ATCC 35405 [30] were used in this study. The MMBC-3 medium with 8 μM FeSO${}_{4}$ and 0.08 μM protoporphyrin IX (PPIX) [31] was used for growing *S. gordonii* and *P. gingivalis*, while *T. denticola* was first cultured in the NOS spirochete medium [32] and further sub-cultured in the MMBC-3 with FeSO${}_{4}$ and PPIX. The three microorganisms were grown in anaerobic condition at 37 ${}^{\circ }$C in an anaerobic chamber (MACS 500, Don Whitley Scientific, Bingley, UK) with 10% v:v H${}_{2}$, 10% v:v CO${}_{2}$, and 80% v:v N${}_{2}$.

#### 2.2.2. Bacterial Growth Conditions to Assess Initial Attachment

Two hundred μL of filtered (0.20 μm) and twice-diluted (in sterile water) saliva (Pool Human Donors, MyBioSource) was used to coat an eight-chambered polymer coverslip/μ-slide (ibiTreat, Ibidi) for 30 min. The saliva was replaced with 200 μL of the inocula consisting of *S. gordonii* (OD${}_{600nm}$ = 0.05) and/or *P. gingivalis* (OD${}_{600nm}$ = 0.1) and/or *T. denticola* (OD${}_{600nm}$ = 0.1) alone and in different combinations to have mono-species, dual-species, and three-species cultures at three iron concentrations (0.8 or 8 or 80 μM FeSO${}_{4}$ with 0.08 μM PPIX). These culture-containing μ-slides were incubated in anaerobic condition for 2 h to enable the process of initial bacterial attachment and biofilm initiation in each well of the slide. After 2 h, the planktonic cells were removed and the μ-slides containing attached sessile cells were washed with 200 μL of PBS. These sessile bacterial cells (obtained post 2 h of incubation) were used for microscopic imaging and for bacterial species quantification.

#### 2.2.3. Confocal Laser Microscopy and Imaging

The sessile bacterial cells (obtained post 2 h of incubation) grown at three iron levels (0.8 or 8 or 80 μM FeSO${}_{4}$ with 0.08 μM PPIX) were stained using the Syto^®^9 (5 μM) green-fluorescent nucleic acid stain (Invitrogen, ThermoFisher Scientific) diluted in PBS and incubated for 15 min. These stained cells were observed *in situ* with a Leica TCS-SP5 confocal laser scanning microscope (Leica Microsystems, Wetzlar, Germany). Using an HC PL Apo 63X, 1.4 NA, oil immersion objective lens, images were captured. However, mono-species sessile cells of *T. denticola* alone displayed scanty cells and a magnification of 63X focused either on only the bacteria or only void spaces, giving biased images. Hence, a magnification of 40X was used using an HC PL Apo 40X, 1.25-0.75 NA, oil immersion lens to capture clear images of the spirochete. A numerical zoom of 1.5 was applied to all acquisitions. All Syto^®^9-stained bacteria were detected using the 488 nm UV diode and a 485 to 500 nm band-pass emission filter. Biofilm stacks (123 × 123 μm) acquired at 1 μm intervals were scanned with a line average of 2. Leica software (LAS AF V.2.2.1) was used for microscope piloting and image acquisition.

Image analyses were performed using the Comstat2 plugin in the ImageJ software V1.43m (National Institute of Health, Edmond, OK, USA) to estimate characteristic microscopy parameters: the biovolume (the volume occupied by the microcolonies divided by the surface end expressed in μm${}^{3}$/μm${}^{2}$) representing the overall volume of the microcolonies, the mean thickness (μm) on all the surface and the mean thickness on bacteria (μm) without the void surface, roughness coefficient, which is calculated from the thickness distribution and is an indicator of the heterogeneity, the surface-to-volume ratio (μm${}^{2}$/μm${}^{3}$, surface of the microcolonies divided by their volume), and the maximum thickness (μm). All the parameters are described in [33]. After microscopic observation, the stain was removed from the μ-slides’ wells and the attached 2 h cells were collected in 100 μL of PBS, centrifuged (8000× *g*, 10 min, 20 ${}^{\circ }$C), and the pellets were stored at −20 ${}^{\circ }$C for further characterization by qPCR. Three-dimensional images were generated with Imaris Viewer 9.6 software from biofilm stack obtained using the Leica TCS-SP5 confocal laser scanning microscope.

#### 2.2.4. qPCR Quantification

The stored pellets (at −20 ${}^{\circ }$C) of the sessile *S. gordonii*, *P. gingivalis*, and/or *T. denticola*, along with stored pellets of 200 μL of the inocula used for each condition (centrifuged and stored as pellets at −20 ${}^{\circ }$C), were resuspended in 100 μL of PBS. These were further heated for 20 min at 95 ${}^{\circ }$C. The concentrations of the DNA in these samples were determined by performing quantitative PCR against defined concentrations of DNA standards set in the range of 0.0001 to 10 ng with purified genomic DNA from each of the three species. A total reaction volume of 12.5 μL contained 6.25 μL SYBR 2X Green Master Mix (Eurogentec, Seraing, Belgium), 1 μL each of forward and reverse primers (5 μM), and 1 μL of DNA template. DNA templates were amplified using the Applied Biosystems apparatus (StepOne Plus, Waltham, MA, USA). The conditions for qPCR were as follows: an initial holding stage of 95 ${}^{\circ }$C for 10 min followed by 40 cycles of 15 s at 95 ${}^{\circ }$C and 1 min at 60 ${}^{\circ }$C; a melt curve stage was performed consisting of 15 s at 95 ${}^{\circ }$C followed by a temperature gradient from 60 ${}^{\circ }$C to 95 ${}^{\circ }$C in 1 ${}^{\circ }$C increment steps, measuring fluorescence at each temperature for 15 s. Primers used were specific to each species targeting the 16S ribosomal RNA, taking into account specific genome weights [9,34]. The primers used in this study are listed in Table S1 in Supplementary Materials.

#### 2.2.5. Statistical Analysis

All the experiments were carried out with a minimum of two biological and two technical replicates (n > 4). Statistical analysis was performed using the two-tailed unpaired Student’s t-test, and a *p*-value of less than 0.05 was considered statistically significant.

## 3. Results

### 3.1. Characteristics of the Attachment Model

**Figure 2:**
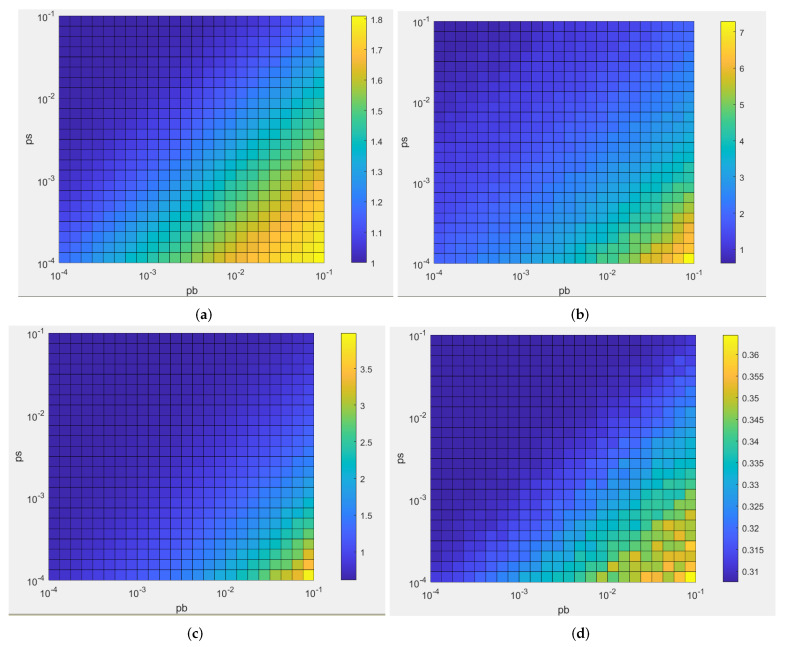
Effect of the ratio of probability ps values against pb values on bacterial attachment characteristics using 2D one-species model for (**a**) roughness coefficient, (**b**) maximum thickness, (**c**) mean thickness on bacteria only, (**d**) mean thickness on the whole surface (including voids). A total of 50 simulations were performed with $Nb_{cell}=100$ and $pb=pb_{1}=pb_{2}$. Color grade indicates the average value of each bacterial attachment criterium according to pb or ps values.

**Figure 3:**
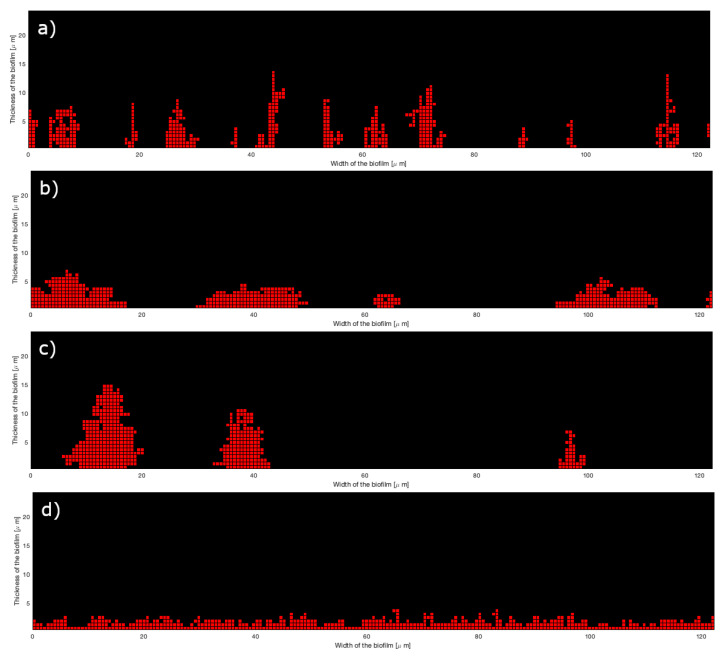
A 2D simulation of attachment for $Nb_{cell}=500$ with (**a**) $\left(ps,\;pb_{1},\;pb_{2}\right)=\left(0.001,\;0.05,\;0.2\right)$, (**b**) $\left(ps,\;pb_{1},\;pb_{2}\right)=\left(0.001,\;0.2,\;0.05\right)$, (**c**) $\left(ps,\;pb_{1},\;pb_{2}\right)=\left(0.0001,\;0.1,\;0.1\right)$, (**d**) $\left(ps,\;pb_{1},\;pb_{2}\right)=\left(0.1,\;0.0001,\;0.0001\right)$.

An extensive sensitivity analysis was performed to evaluate the effect of the algorithm parameters (see Figure 2, Figures S1 and S2 in Supplementary Materials). It is not the specific value but the ratios between the three parameters ps, pb1, and pb2 that are important. As shown in Figure 2, for each bacterial attachment criterium (roughness, maximum and mean thicknesses), ratio of pb/ps determines the level of intensity of each criterium, with high values of bacterial attachment criteria for high pb/ps ratios. Moreover, as expected and as shown in Figure 3, which represents 2D simulations of mono-bacterial biofilms with different probabilities values:

- For an identical ps value (Figure 3a,b), if horizontal bacterial adhesion probability pb1 is smaller than vertical probability pb2 (Figure 3a), then microcolonies are more vertically extended. In the reverse case (Figure 3b), microcolonies are horizontally extended.
- If surface attachment probability is much smaller than bacterial attachment probabilities (ps<pb1 and pb2), few microcolonies develop (see Figure 3c), otherwise many microcolonies can cover the whole surface (see Figure 3d).

Thus, different sets of values of parameters with the same ratios lead to the same kind of microcolonies with similar statistical characteristics.

Moreover, the model being stochastic, two simulations with the same value of parameters do not give exactly the same result measured by the statistical variables. When the algorithm is run 10,000 times with the same values of parameters, the results for the roughness coefficient follow a normal distribution, whereas the distribution for the mean thickness or the mean thickness on biofilm is asymmetric. It is a generalized extreme value distribution. See Figure S2 in Supplementary Materials.

**Figure 4:**
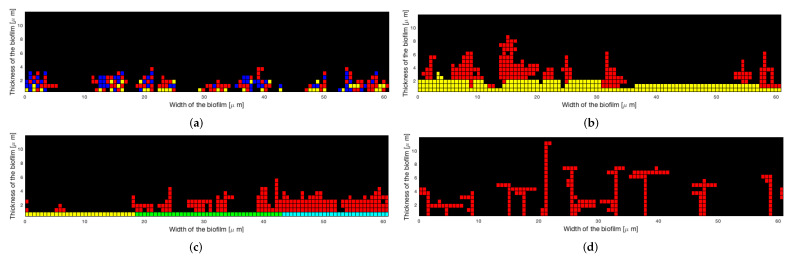
Two-dimensional illustrations of algorithm extensions for $Nb_{cell}=200$: (**a**) Attachment of three species of bacteria with 40 (yellow), 60 (blue), and 100 (red) elements, respectively, ps=[0.05,0.001,0.00001] and other probabilities equal 0.1. (**b**) Attachment on a non-plane surface with ps=0.001, $pb_{1}=pb_{2}=0.1$. (**c**) Attachment on an inhomogeneous plane surface with ps=0.001 at the left, 0.005 in the middle, 0.05 at the right, $pb_{1}=pb_{2}=0.05$. (**d**) Attachment with ps=0.001 and variable values for $pb_{1}$ and $pb_{2}$: $pb_{1}=0.01$ and $pb_{2}=0.2$ for the 100 first elements, and then $pb_{1}=0.2$ and $pb_{2}=0.01$.

An illustration of the previously presented extensions can be found in Figure 4 and in Section 3.3 for the 3D model.

### 3.2. Biological Characterization of Biofilms Initiation by Oral Bacteria

Since the systemic iron overload disease hemochromatosis has been previously associated with chronic periodontitis, we investigated the effect of iron levels and interspecies associations between an oral commensal *S. gordonii* and two periodontal pathogens *P. gingivalis* and *T. denticola* in biofilm initiation. Three iron concentrations have been tested (0.8, 8, and 80 μM), 8 μM being the optimal concentration for the growth of these species. The ability of these species to form mono-, dual-, and three-species 2 h microcolonies at different iron levels was evaluated using quantitative PCR and confocal microscopy.

#### 3.2.1. Effect of Iron Levels and Interspecies Associations on Initial Structure

**Figure 5:**
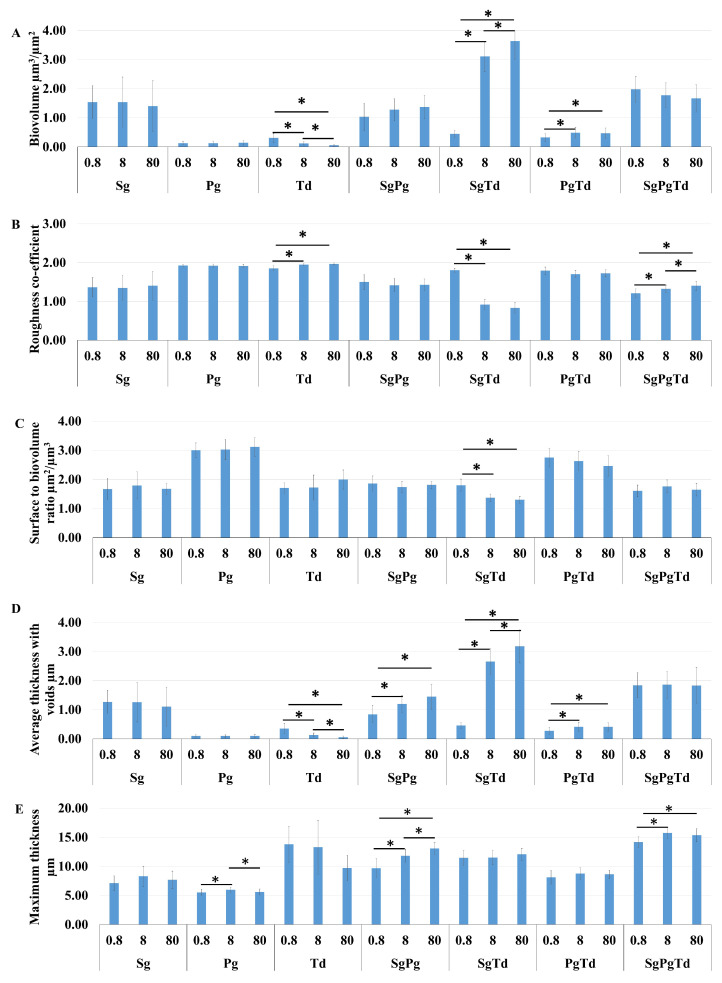
Effect of iron on the microscopic parameters obtained for mono-, dual- and three-species sessile cells. Effect of 0.8 μM, 8 μM, and 80 μM of iron on the (**A**) biovolume, (**B**) roughness coefficient, (**C**) surface-to-biovolume ratio, (**D**) mean thickness, and (**E**) maximum thickness of mono-species (*S. gordonii*—Sg, *P. gingivalis*—Pg, and *T. denticola*—Td), dual-species (*S. gordonii*-*P. gingivalis*: SgPg, *S. gordonii*-*T. denticola*: SgTd, *P. gingivalis*-*T. denticola*: PgTd), and three-species (*S. gordonii*-*P. gingivalis*-*T. denticola*: SgPgTd) 2 h sessile growth. The microscopic parameters were calculated on the total bacteria in each condition (comprising all cells irrespective of individual species). * indicates *p*-value < 0.05.

**Figure 6:**
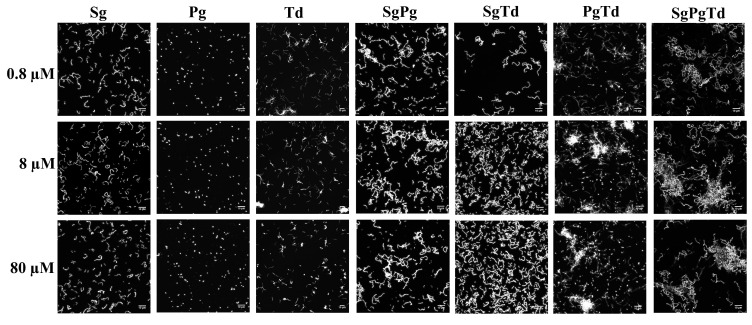
Representative microscopic images of bacteria attached at 2 h in mono-, two-, and three-species conditions. The 2 h sessile cells of various conditions (mono-species: *S. gordonii*—Sg, *P. gingivalis*—Pg, *T. denticola*—Td; dual-species: *S. gordonii*-*P. gingivalis*: SgPg, *S. gordonii*-*T. denticola*: SgTd, *P. gingivalis*-*T. denticola*: PgTd; three-species: *S. gordonii*-*P. gingivalis*-*T. denticola*: SgPgTd) were stained using Styo^®^9 and were visualized using the Leica TCS-SP5 confocal laser scanning microscope. The images are representative of the total bacteria in each condition (comprising all cells irrespective of individual species). The images of these 2 h sessile cells grown at three different iron concentrations (0.8 μM, 8 μM, and 80 μM) were compared. A maximum z-projection of the Z stack was taken using 40× oil immersion objective lens for *T. denticola* mono-species condition while the 63× oil immersion objective lens was used for the remaining. A numerical zoom of 2 was applied. The scale (10 μm) is shown on the bottom right corner of each image.

**Figure 7:**
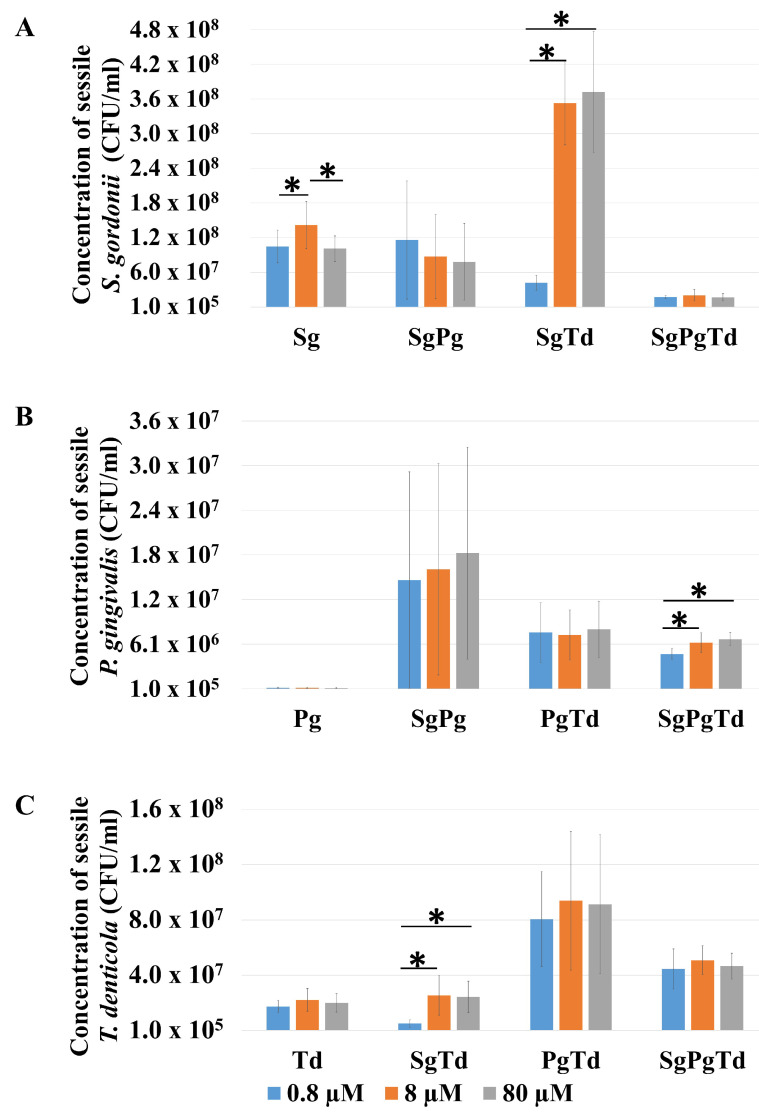
Effect of iron and interspecies association on attachment of each species in either mono- or multi-species sessile cells. The concentration of individual species (CFU/mL) in different conditions (mono-species: *S. gordonii*-Sg, *P. gingivalis*-Pg and *T. denticola*-Td; dual-species: *S. gordonii*-*P. gingivalis*: SgPg, *S. gordonii*-*T. denticola*: SgTd, *P. gingivalis*-*T. denticola*: PgTd; three-species: *S. gordonii*-*P. gingivalis*-*T. denticola*: SgPgTd) were compared at 0.8 μM, 8 μM, and 80 μM of iron. The graph shows the concentration of *S. gordonii* (**A**), concentration of *P. gingivalis* (**B**), and concentration of *T. denticola* (**C**) in the different 2 h sessile cells. All initial inoculums contained 2.8 × 10${}^{8}$ cells of *S. gordonii* and/or 1.2 × 10${}^{9}$ cells of *P. gingivalis* and/or 6.3 × 10${}^{8}$ cells of *T. denticola* in the various conditions. * indicates *p*-value < 0.05.

In the mono-species condition, the 2 h *P. gingivalis* and *T. denticola* sessile cells showed low values for biovolume and mean thicknesses (Figure 5A,D) in comparison to *S. gordonii*. This result is consistent with the negligible initial attachment of *P. gingivalis* and *T. denticola* as shown in microscopic images (Figure 6) and measured by qPCR (Figure 7B, Figure S3 in Supplementary Materials). Moreover, *P. gingivalis* and *T. denticola* mono-species were heterogeneous (demonstrated by an elevated roughness coefficient) (Figure 5B). The surface to biovolume ratio of *P. gingivalis* was the highest among the mono-species condition (Figure 5C).

For the attached dual-species *S. gordonii*-*P. gingivalis* cells, the values for biovolume, roughness coefficient, and surface to biovolume ratio showed no change due to iron concentration and were similar to that of mono-species *S. gordonii* (Figure 5A–C), perhaps due to the higher proportion of *S. gordonii* (Figure 7A,B). The microscopy results were in concurrence with the results of cell concentrations measured by qPCR which also remained unaffected by iron levels. However, the thickness (average and maximum) significantly increased at high iron concentrations (Figure 5D,E), suggesting an effect on the initial biofilm architecture.

In the case of attached dual-species *S. gordonii*-*T. denticola* cells, the biovolume (Figure 5A) increased at 8 and 80 μM of iron compared to 0.8 μM, in accordance with qPCR data (Figure S3 and Figure 7A,C). Moreover, a significantly lower roughness coefficient and surface to biovolume ratio for dual-species *S. gordonii*-*T. denticola* sessile cells (Figure 5B,C) can be attributed to the more homogenous distribution of these bacteria across the surface (Figure 6). The mean thickness of dual-species *S. gordonii*-*T. denticola* increased with iron levels while its maximum thickness remained unaffected by iron concentration (Figure 5D,E).

For the attached dual-species *P. gingivalis*-*T. denticola* cells, the biovolume was significantly higher at 8 and 80 μM of iron when compared to 0.8 μM (Figure 5A and Figure 6). The mean thickness of the dual-species was low, corresponding to the low thickness of each species in the mono-species condition (Figure 5D).

In the case of three-species attached bacterial cells, the maximum thickness was higher at 8 and 80 μM of iron than at 0.8 μM (Figure 5E). The roughness coefficient of the attached three-species cells was high irrespective of iron levels and increased with the concentration of iron (Figure 5B). The high roughness coefficient at all iron levels may be due to the extensive clustering of bacteria (Figure 6) observed in the three-species condition resulting in unevenly distributed growth on the substratum. The values of the microscopic experimental measurements can be found in Supplementary Material (see Table S2).

#### 3.2.2. Effect of Iron and Interspecies Association on Attachment of Each Species in Mono and Multi-Species Conditions

The effect of iron on the biofilm initiation ability of individual species in mono, dual-, and three-species sessile growth was analyzed and compared. In pure cultures, 8 μM of iron favored *S. gordonii* attachment compared to 0.8 μM of iron and 80 μM of iron, while no difference was observed for *P. gingivalis* and *T. denticola* (Figure 7). Attached *S. gordonii* levels were comparable in mono-species and dual-species sessile growth with *P. gingivalis*. In contrast, attachment of *S. gordonii* cells was promoted by the presence of *T. denticola* at 8 or 80 μM of iron and reduced at 0.8 μM, compared to mono-species conditions (Figure 7A). Among all conditions (mono-, dual-, and three-species), the concentration of *S. gordonii* was the lowest in the case of three-species condition irrespective of iron level. This may imply a detrimental effect of *P. gingivalis* and *T. denticola* when together on *S. gordonii* development in the early biofilm.

In the case of *P. gingivalis*, very few cells were attached in mono-species conditions (Figure 7B). The presence of *S. gordonii* and/or *T. denticola* significantly increased the levels of *P. gingivalis* in attached bacterial cells, even if at a lower rate with *T. denticola*, independently of the iron concentration used (Figure 7B and Table S3). Finally, *P. gingivalis* attachment in three-species condition was lower than in dual-species of either *S. gordonii*-*P. gingivalis* or *P. gingivalis*-*T. denticola*. It seems that the positive individual effect of both *S. gordonii* and *T. denticola* on *P. gingivalis* attachment was reduced when they were together in the inoculum. Iron displayed no effect on *P. gingivalis* attachment, except in the three-species condition, with a positive effect at higher iron levels (8 and 80 μM) (Figure 7B).

In the case of *T. denticola* (Figure 7C and Table S3), highest concentrations were observed in the presence of *P. gingivalis*, which suggested a beneficial effect of *P. gingivalis* on *T. denticola* at all iron levels. The lowest concentration of *T. denticola* was observed in either mono-species or in dual-species conditions with *S. gordonii* with no significant difference between them. In the three-species condition, *S. gordonii* and *P. gingivalis* together favored the attachment of *T. denticola* when compared to mono-species. Interestingly, the proportion of *T. denticola* cells (approximately 65%) was the highest (with *S. gordonii*—27%, *P. gingivalis*—8% approximately) in the three-species sessile growth at all iron levels. However, the attachment of *T. denticola* in the three-species condition was lower than that of dual-species *P. gingivalis*-*T. denticola* for all iron concentrations. These data suggest that the positive effect of *P. gingivalis* on *T. denticola* attachment was reduced when *S. gordonii* was also present. Iron affected the levels of *T. denticola* only in the *S. gordonii*-*T. denticola* sessile growth, favoring increased levels at 8 and 80 μM as compared with 0.8 μM iron. *S. gordonii* at low iron level displayed a detrimental effect on *T. denticola* development compared to *T. denticola* in mono-species condition.

### 3.3. Simulation of Oral Bacterial Attachment

**Figure 8:**
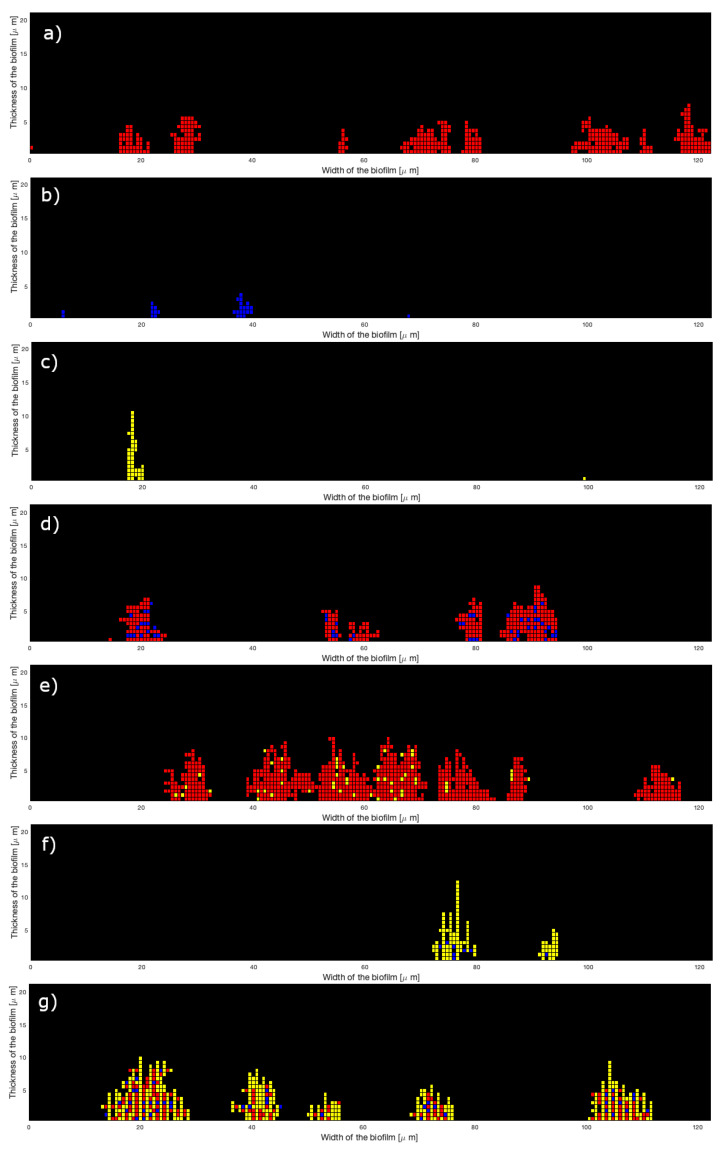
Two-dimensional simulations of biofilms attachment at 8 μM of iron: (**a**) Sg (red), (**b**) Pg (blue), (**c**) Td (yellow), (**d**) SgPg, (**e**) SgTd, (**f**) PgTd, (**g**) SgPgTd.

**Figure 9:**
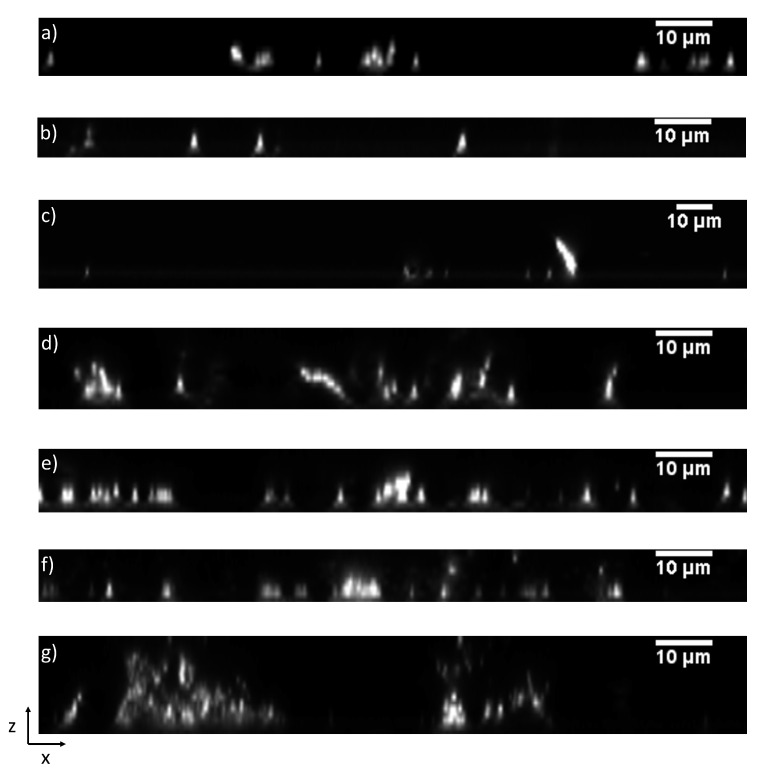
The 2D slices, generated with ImageJ software V1.43m from biofilm stacks obtained using Leica TCS-SP5 confocal laser scanning microscope, and obtained at 8 μM iron for (**a**) Sg, (**b**) Pg, (**c**) Td, (**d**) SgPg, (**e**) SgTd, (**f**) PgTd, (**g**) SgPgTd. Their respective X–Z projection or thickness (z) along the X axis is shown. The scale (10 μm) is shown on the top right corner of each image.

The three parameters ps (probability of attachment on the surface), pb1 (horizontal attachment probability on bacteria), and pb2 (vertical attachment probability on bacteria) were first fitted for each species of bacteria using the results of the corresponding experiments for one species of bacteria alone. Then, the results of experiments for two species of bacteria were used to fit the interspecies parameters pbi1 (horizontal attachment probability between the two species of bacteria) and pbi2 (vertical attachment probability between the two species of bacteria) by fixing the parameters ps, pb1, and pb2 to the previous fitted values. Finally, the attachment of the three species of bacteria was simulated with the parameters’ values fixed previously and the results were compared to the experimental data. To compare the results of simulation with the experimental results, we denote $E_{biovol}$, $E_{hmean}$, $E_{rough}$, $E_{hmeanb}$, $E_{hmax}$ the relative errors defined by

$$E_{X}=\frac{|X-X_{exp}|}{X_{exp}}$$

where *X* is the biovolume (biovol), the mean thickness (hmean), the roughness coefficient (rough), the mean thickness on bacteria (hmeanb), or the maximum thickness (hmax), and $X_{exp}$ the corresponding experimental data. Table 1, Table 2 and Table 3 give the fitted values of the parameters and the relative errors for the 2D model at 0.8, 8, and 80 μM of iron, respectively. To easily compare the results, the value of pb2 has been fixed to 0.10 for the three species of mono-bacterial colonies (because it is the ratios between the three parameters that has an effect on the structure; see Section 3.1). Sometimes the results of experiments are not precise enough and the measured biovolume is higher than the measured mean thickness (see Tables S3 and S4 in Supplementary Materials). This is probably due to inaccuracies in biological measurements on very weak data. In this case, we chose to better fit the mean thickness instead of the biovolume; therefore, the relative error is always low for the mean thickness but not for the biovolume. The biovolume is directly dependent on the number $Nb_{cell}$ of cells of the grid filled during the attachment: it is the product of the cell volume by $Nb_{cell}$. Figure 8 presents an example of simulation results for each of the seven kinds of microcolonies for the 2D model at 8 μM of iron. It can be compared to 2D experimental images of Figure 9. As shown in Table 1, Table 2 and Table 3, the relative errors between the experimental and simulated results are low for the three-species microcolonies except for 0.8 μM of iron.

**Figure 10:**
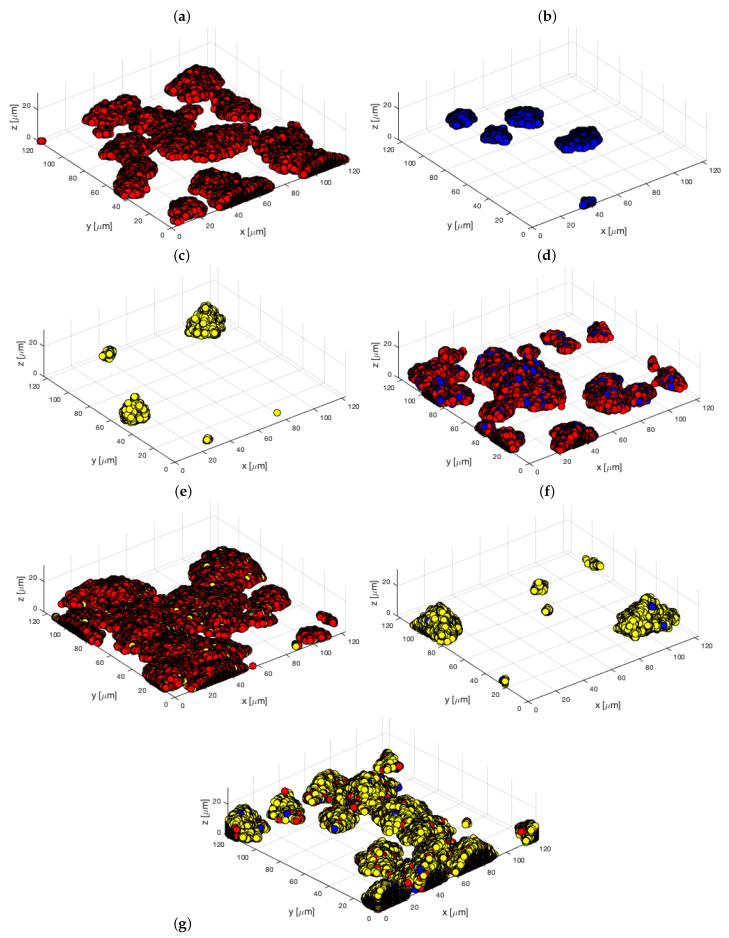
Three-dimensional simulations of biofilms attachment at 8 μM of iron: (**a**) Sg (red), (**b**) Pg (blue), (**c**) Td (yellow), (**d**) SgPg, (**e**) SgTd, (**f**) PgTd, (**g**) SgPgTd.

**Figure 11:**
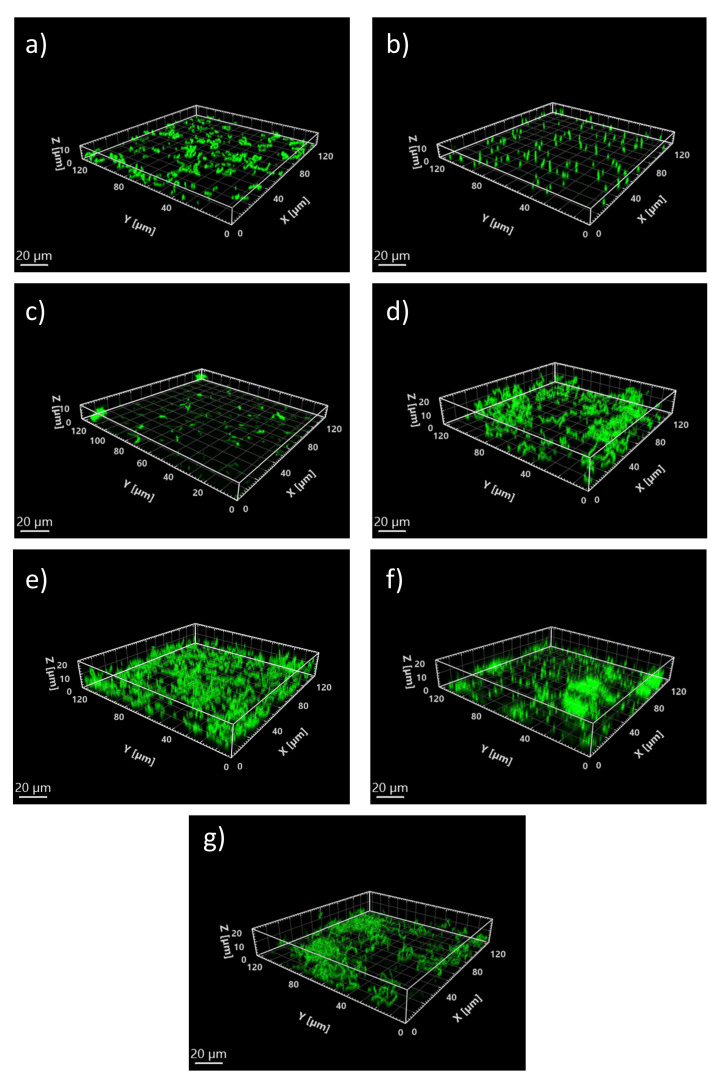
Representative 3D images generated with Imaris Viewer 9.6 software from biofilm stacks obtained using Leica TCS-SP5 confocal laser scanning microscope, and obtained at 8 μM iron for (**a**) Sg, (**b**) Pg, (**c**) Td, (**d**) SgPg, (**e**) SgTd, (**f**) PgTd, (**g**) SgPgTd. The scale (20 μm) is shown on the bottom left corner of each image.

The same process was applied to the 3D model. The results are given in Table 4 and Figure 10 for 8 μM of iron. These results can be compared to 3D experimental images of Figure 11 and numerical values in Table S4.

The attachment capabilities of different species of bacteria can be compared using the ratios between the parameters of mono-bacterial microcolonies, given in Table 5.

## 4. Discussion

### 4.1. Dependence of the Characteristics of the Microcolonies on the Algorithm Parameters

We present first some remarks on the choice of the parameters’ values to obtain specific microcolonies for the 2D model. These remarks can be generalized to the 3D model.

When the domain and dx are fixed, the biovolume biovol of the microcolonies depends only on $Nb_{cell}$: $biovol=Nb_{cell}dx/N_{x}$.

If there was no void below the top of the microcolonies (for example, in using the option of the algorithm to avoid voids), the mean thickness hmean would be equal to the biovolume and depend only on $Nb_{cells}$. Then, hmean is expected to be always superior to biovol and the difference, hmean−biovol, is an indicator of the presence of void inside the microcolonies. hmean also depends on the ratios $\frac{ps}{pb1}$ and $\frac{pb1}{pb2}$. If ps is small compared to pb1 and pb2, there will be few microcolonies that will favor the void inside the microcolonies, otherwise the microcolonies will be numerous and will tend to cover all the substratum. The void inside the microcolonies is also favored by a ratio $\frac{pb1}{pb2}$ around 1: if pb1 is small compared to pb2, the microcolonies grow taller, and with pb2 small compared to pb1, they grow wider but with little void in both cases.

The roughness coefficient depends on $Nb_{cells}$ and the ratios of the parameters but, principally, it increases when $\frac{pb1}{pb2}$ or $\frac{ps}{pb1}$ decreases.

The mean thickness on bacteria hmeanb have the same dependencies as hmean but it is always superior to hmean. It is also more sensitive to the ratio $\frac{pb1}{pb2}$ if the microcolonies do not cover all the substratum. If they cover all the substratum, hmeanb=hmean.

The maximum thickness hmax has dependencies similar to that of hmeanb but is very dependent on the ratio $\frac{pb1}{pb2}$: hmax increases when $\frac{pb1}{pb2}$ decreases.

For pb=pb1=pb2, the characteristics of the microcolonies (roughness, mean thickness, mean thickness on bacteria, maximum thickness) are similar when the ratio $\frac{ps}{pb}$ is constant. Moreover, the values increase when the ratio decreases, especially the roughness coefficient. For a large ratio, the structure is flat and covers the surface as in Figure 3d, but for a small ratio, there are few higher microcolonies, as in Figure 3c. Thus, a bacterium that has a larger ratio than another has a better capability to attach to the surface.

If ps is fixed, the roughness coefficient, the mean thickness on bacteria, and the maximum thickness increase when the ratio $\frac{pb1}{pb2}$ decreases but the effect on the mean thickness is comparatively small. For a small ratio, the microcolonies are tall, as in Figure 3a, but for a large ratio the microcolonies are lower and spread horizontally, as in Figure 3b. The number of microcolonies is greater in Figure 3a because the ratio $\frac{ps}{pb1}$ is smaller.

The illustrations presented in Figure 4 show the ability of the algorithm to be used in multiple situations. The probability of attachment on the surface ps can be used to distinguish different materials (see Figure 4c) or different species of bacteria, as in Figure 4a, where the least numerous type of bacteria (yellow) is the most present on the surface because of the highest value of ps. It is a way to characterize the primary colonizers.

### 4.2. Ability of the Model to Fit the Experimental Oral Microcolonies

From Table 1, Table 2, Table 3 and Table 4 and Figure 4 and Figure 10, we can deduce that the algorithm can create realistic microcolonies of oral bacteria *S. gordonii*, *P. gingivalis*, and *T. denticola* with similar characteristics to the experimental microcolonies. An important relative error on the biovolume appears when there is an inconsistency of the experimental data with a biovolume greater than the mean thickness.

The ratios of the parameters are presented in Table 5. The more important value of the ratio $\frac{ps}{pb}$ is obtained for *S. gordonii* which is a commensal species. The ratio $\frac{pb1}{pb2}$ is also higher for *S. gordonii* with microcolonies flatter and less high than for *T. denticola*.

For mono-bacterial experiments, at 8 μM iron, best fits were obtained (with the smallest errors between simulated and experimental models) with probability of surface attachment being the highest for *S. gordonii*, and lower but identical for the two other species. This is well in agreement with primary colonizer properties of the *Streptococcus* species. Variations of iron concentrations at lower or higher values did not modify the ps values for *S. gordonii* and *P. gingivalis*. In contrast, ps values obtained for *T. denticola* with best fits vary with iron concentrations: ps probability decreases with iron increase in the medium. Indeed, experimental *T. denticola* microscopy results displayed variations according to iron levels, with thickness being decreased with iron increase.

Regarding inter-cells bacterial attachment, to obtain the best consistency between experimental and mathematical values, it was necessary to set vertical and horizontal attachment probabilities pb1 and pb2 to identical values for the *Streptococcus* species. This means that this species would attach to its counterparts in any dimensional direction. For the two other species, it was required to set one of the bacterial attachment probabilities to lower values than the other dimensional probability (horizontal probability of attachment were lower than vertical ones in the simulations described), with the lowest values obtained for *T. denticola*. This could be explained by the non-symmetrical shape of these two species, which can influence adhesion between bacteria. Overall, pb values for these two species were lower than *S. gordonii* pb values, corresponding to lower potential of intraspecies attachment for these species.

For dual-species experiments (intraspecies probability values being fixed using mono-species simulations), fitting of interspecies attachment probabilities depends on the nature of the two species present in inocula. At 8 μM iron:

- For both *T. denticola*-containing inocula (SgTd and PgTd), the lowest errors were obtained by setting horizontal probabilities at the highest value, whereas the vertical probability was 250 times less. This would mean that bacteria attach predominantly next to other bacteria and less on top of them.
- The reverse was observed with SgPg-containing inocula, with a vertical probability higher than the horizontal one for best fitting. Overall, horizontal and vertical probabilities for this type of species interaction are higher, suggesting that *S. gordonii* and *P. gingivalis* would attach better together than the other types of species.

Iron levels did not have major effects on PgTd and SgPg interactions, as probabilities are quite similar at 0.8, 8, and 80 μM. The same is true for SgTd interactions between 8 and 80 μM. However, when iron was decreased to 0.8 μM, horizontal and vertical probabilities values were completely inverted, with low values for the horizontal component and high value for vertical component. There seems to be a different attachment process between the two iron concentrations, which is consistent with low levels of bacteria attached and low thicknesses observed for this interaction type.

When these two-species interspecies probabilities were applied to the three-species model, a very good fit is obtained between the simulation and the experiment for 8 μM and 80 μM of iron. It shows that the attachment characteristics of each species of bacteria and the interactions between them are well described by the values of the parameters. For 0.8 μM, the results are not so good, probably because the interaction between *S. gordonii* and *T. denticola* is not well approached: for example, if we exchange pbi1 and pbi2 for SgTd, the results become better for SgPgTd but worse for SgTd. Another explanation could be the effect of a new interaction between the three species.

Regarding the three-dimensional modeling of attachment at 8 μM iron, interspecies probabilities of attachment were similarly fitted, except for SgTd interactions for which the vertical component was 20 times higher in 3D compared with 2D. The mean thickness on bacteria and the maximum thickness are better approached by the 3D model, because a greater number of filled cells allows a greater thickness while respecting the mean height and the roughness coefficient.

In the process of the algorithm, only one cell of the grid is filled at a time. It does not simulate the attachment of aggregations of bacteria that cannot be excluded. Experimental data on this subject is not available and is difficult to obtain, but the good fit of results between numerical simulation and experimentation shows that the attachment of bacterial aggregates is not preponderant or has not an important effect on the statistical characteristics of the microcolonies.

## 5. Conclusions

A stochastic model of bacterial adhesion to a surface was developed and evaluated. Its ability to simulate real attachments characterized by statistical data was validated by comparison with experimental data on *P. gingivalis*, *S. gordonii*, and *T. denticola*. The ratios between three parameters, ps, pb1, and pb2 (probability of attachment to the surface or to the horizontal side of the bacterial cells or to the vertical side of the cells respectively), appeared as the key parameters for the simulation of realistic attachments of bacteria. Guidance on how to use the model was given in various situations.

This model and its comparison with experimental data made it possible to highlight attachment characteristics linked to bacterial species and iron concentration. If attachment and growth are simultaneous, this algorithm can be coupled simultaneously with a growth model. It is also a useful tool to build initial realistic microcolonies for a biofilm growth simulation. The next step of our study will be to use this attachment model for the initiation of a biofilm growth model to analyze the development of oral biofilms at different iron concentrations with the same three bacterial species.

## Figures and Tables

**Table 1:** Two-dimensional oral bacterial attachment model fitting using experimental biological values at 0.8 μM of iron. Relative errors are computed on the mean of 100 simulations (0 represents a value less than 0.005).

Bacteria	${\mathrm{Nb}}_{\mathrm{cell}}$	ps	pb1	pb2	$E_{\mathrm{biovol}}$	$E_{\mathrm{hmean}}$	$E_{\mathrm{rough}}$	$E_{\mathrm{hmeanb}}$	$E_{\mathrm{hmax}}$
Sg	365	$7\times 10^{-4}$	0.10	0.10	0.27	0.01	0.03	0.08	0.16
Pg	29	$1.5\times 10^{-4}$	0.06	0.10	0.31	0.01	0	0.03	0.21
Td	99	$1.5\times 10^{-4}$	0.01	0.10	0.02	0.02	0	0.05	0.12
		pbi1	pbi2						
SgPg	240	0.05	0.20		0.28	0.02	0.01	0.02	0.18
SgTd	126	0.001	0.25		0.12	0	0.07	0.40	0.45
PgTd	75	0.25	0.001		0.28	0.01	0.03	0.35	0.02
SgPgTd	500				0.23	0.06	0.22	0.70	0.37

**Table 2:** Two-dimensional oral bacterial attachment model fitting using experimental biological values at 8 μM of iron. Relative errors are computed on the mean of 100 simulations (0 represents a value less than 0.005).

Bacteria	${\mathrm{Nb}}_{\mathrm{cell}}$	ps	pb1	pb2	$E_{\mathrm{biovol}}$	$E_{\mathrm{hmean}}$	$E_{\mathrm{rough}}$	$E_{\mathrm{hmeanb}}$	$E_{\mathrm{hmax}}$
Sg	360	$7\times 10^{-4}$	0.10	0.10	0.28	0	0.02	0.03	0
Pg	28	$1.5\times 10^{-4}$	0.06	0.10	0.34	0	0	0.04	0.29
Td	39	$5\times 10^{-5}$	0.01	0.10	0	0.03	0	0.02	0.30
		pbi1	pbi2						
SgPg	340	0.05	0.20		0.18	0.02	0	0	0.20
SgTd	800	0.25	0.001		0.21	0.05	0.06	0.19	0.05
PgTd	110	0.25	0.001		0.31	0.01	0.08	1.00	0.34
SgPgTd	560				0.03	0.02	0.01	0.05	0.27

**Table 3:** Two-dimensional oral bacterial attachment model fitting using experimental biological values at 80 μM of iron. Relative errors are computed on the mean of 100 simulations (0 represents a value less than 0.005).

Bacteria	${\mathrm{Nb}}_{\mathrm{cell}}$	ps	pb1	pb2	$E_{\mathrm{biovol}}$	$E_{\mathrm{hmean}}$	$E_{\mathrm{rough}}$	$E_{\mathrm{hmeanb}}$	$E_{\mathrm{hmax}}$
Sg	315	$7\times 10^{-4}$	0.10	0.10	0.31	0	0.02	0.01	0.02
Pg	29	$1.5\times 10^{-4}$	0.06	0.10	0.36	0.02	0	0.08	0.24
Td	16	$1\times 10^{-6}$	0.01	0.10	0	0	0	0.17	0.31
		pbi1	pbi2						
SgPg	400	0.001	0.25		0.11	0.02	0.04	0.05	0.13
SgTd	970	0.25	0.001		0.18	0.06	0.07	0.20	0.02
PgTd	110	0.25	0.001		0.28	0.01	0.09	1.43	0.57
SgPgTd	545				0	0.01	0.03	0	0.21

**Table 4:** Three-dimensional oral bacterial attachment model fitting using experimental biological values at 8 μM of iron. Relative errors are computed on the mean of 100 simulations (0 represents a value less than 0.005).

Bacteria	${\mathrm{Nb}}_{\mathrm{cell}}$	ps	pb1	pb2	$E_{\mathrm{biovol}}$	$E_{\mathrm{hmean}}$	$E_{\mathrm{rough}}$	$E_{\mathrm{hmeanb}}$	$E_{\mathrm{hmax}}$
Sg	78,000	$1\times 10^{-5}$	0.20	0.03	0.22	0.01	0.08	0.10	0.12
Pg	5900	$1.5\times 10^{-6}$	0.20	0.03	0.30	0.01	0	0.05	0.07
Td	7300	$1\times 10^{-6}$	0.08	0.10	0.06	0.02	0	0.09	0.06
		pbi1	pbi2						
SgPg	70,000	0.05	0.20		0.16	0	0	0.04	0.07
SgTd	166,000	0.2	0.02		0.18	0.01	0.03	0.01	0.02
PgTd	24,000	0.25	0.001		0.25	0.01	0.08	0.90	0.90
SgPgTd	114,000				0.01	0.02	0.01	0.06	0.06

**Table 5:** Ratios between attachment parameters for mono-bacterial microcolonies.

Iron Concentration and Model	Bacteria	$\frac{\mathrm{ps}}{\mathrm{pb}1}$	$\frac{\mathrm{ps}}{\mathrm{pb}2}$	$\frac{\mathrm{pb}1}{\mathrm{pb}2}$
0.8 μM 2D model	Sg	$7\times 10^{-3}$	$7\times 10^{-3}$	1
	Pg	$2.5\times 10^{-3}$	$1.5\times 10^{-3}$	0.6
	Td	$1.5\times 10^{-2}$	$1.5\times 10^{-3}$	0.1
8 μM 2D model	Sg	$7\times 10^{-3}$	$7\times 10^{-3}$	1
	Pg	$2.5\times 10^{-3}$	$1.5\times 10^{-3}$	0.6
	Td	$5\times 10^{-3}$	$5\times 10^{-4}$	0.1
8 μM 3D model	Sg	$5\times 10^{-5}$	$3.33\times 10^{-4}$	6.67
	Pg	$7.5\times 10^{-6}$	$5\times 10^{-5}$	6.67
	Td	$1.25\times 10^{-5}$	$1\times 10^{-5}$	0.8
80 μM 2D model	Sg	$7\times 10^{-3}$	$7\times 10^{-3}$	1
	Pg	$2.5\times 10^{-3}$	$1.5\times 10^{-3}$	0.6
	Td	$1\times 10^{-4}$	$1\times 10^{-5}$	0.1

## Supplementary Materials

The following are available at https://www.mdpi.com/article/10.3390/microorganisms10040686/s1, Figure S1: Effect of the ratio of probability pb1 values against pb2 values on bacterial attachement characteristics using 2D one species model, Figure S2: Distribution of the values for the rugosity coefficient of normal law, the mean thickness of generalized extreme law, the mean thickness on biofilm of generalized extreme law, the maximum thickness of discrete law, Figure S3: Comparison between mono-, dual- and three-species conditions, Table S1: List of primers used for the study, Table S2: Microscopic experimental measurements of bacterial attachment, Table S3: Concentration of each species (CFU/mL) in mono-species 0.8, 8 and 80 μM of iron, Table S4: Comparison of 2D simulation, 3D simulation and experimental results at 8 μM of iron
