# Supplementary material for: A Multi-Skilled Mathematical Model of Bacterial Attachment in Initiation of Biofilms
Source: Microorganisms. 2022 Mar 23;10(4):686. doi: 10.3390/microorganisms10040686 (PMC9029265; doi:10.3390/microorganisms10040686)
Supplement: Supplementary file 1 [file microorganisms-10-00686-s001.zip › microorganisms-1586416-supplementary.pdf]

A Multi-Skilled Mathematical Model of Bacterial Attachment in Initiation of Biofilms
Supplementary Material

Kanchana Chathoth Louis Fostier Bénédicte Martin Christine Baysse
Fabrice Mahé

Supplementary Material

Organism	Sequence (5' → 3')
Streptococcus gordonii	F: AAG-CAA-CGC-GAA-GAA-CCT-TA
	R: GTC-TCG-CTA-GAG-TGC-CCA-AC
Porphyromonas gingivalis	F: TGG-GTT-TAA-AGG-GTG-CGT-AG
	R: CAA-TCG-GAG-TTC-CTC-GTG-AT
Treponema denticola	F: CGC-GTC-CCA-TTA-GCT-AGT-TG
	R: TTC-TTC-ATT-CAC-ACG-GCG-TC

Table S1: List of primers used for the study.

Iron	Bacteria	*biovol*[μm^3/μm^2]	*hmean*[μm]	*rough*	*hmeanb*[μm]	*hmax*[μm]
0.8 μM	Sg	1.54±0.57	1.27±0.40	1.36±0.25	4.00±0.69	7.11±1.25
	Pg	0.13±0.06	0.10±0.05	1.92±0.03	2.53±0.28	5.54±0.52
	Td	0.31±0.16	0.36±0.18	1.85±0.06	4.83±0.54	13.78±3.14
	SgPg	1.03±0.46	0.84±0.30	1.50±0.19	3.41±0.37	9.69±1.64
	SgTd	0.45±0.12	0.45±0.10	1.80±0.05	4.63±0.51	11.46±1.32
	PgTd	0.32±0.13	0.28±0.13	1.79±0.10	2.68±0.33	8.12±1.19
	SgPgTd	1.98±0.44	1.84±0.44	1.21±0.11	4.32±0.48	14.16±0.94
8 μM	Sg	1.54±0.86	1.26±0.68	1.35±0.33	3.54±0.51	8.31±1.71
	Pg	0.13±0.07	0.10±0.05	1.92±0.04	2.50±0.35	5.98±0.45
	Td	0.12±0.05	0.14±0.06	1.94±0.03	5.17±1.69	13.28±4.64
	SgPg	1.28±0.38	1.20±0.30	1.42±0.17	4.09±0.61	11.83±1.13
	SgTd	3.11±0.53	2.66±0.44	0.92±0.13	4.17±0.28	11.52±1.22
	PgTd	0.49±0.18	0.42±0.15	1.71±0.09	2.84±0.33	8.75±1.02
	SgPgTd	1.77±0.43	1.86±0.46	1.32±0.10	5.14±0.58	15.74±0.96
80 μM	Sg	1.41±0.87	1.11±0.67	1.41±0.37	3.53±0.69	7.68±1.51
	Pg	0.14±0.07	0.10±0.05	1.91±0.04	2.32±0.30	5.60±0.52
	Td	0.05±0.04	0.06±0.04	1.97±0.03	3.96±0.61	9.71±2.19
	SgPg	1.38±0.39	1.45±0.43	1.43±0.16	4.85±0.53	13.09±0.97
	SgTd	3.64±0.64	3.18±0.57	0.83±0.13	4.56±0.33	12.08±1.04
	PgTd	0.47±0.18	0.41±0.14	1.72±0.09	3.01±0.32	8.62±0.73
	SgPgTd	1.67±0.47	1.83±0.62	1.40±0.12	5.74±0.97	15.36±1.13

Table S2: Microscopic experimental measurements of bacterial attachment

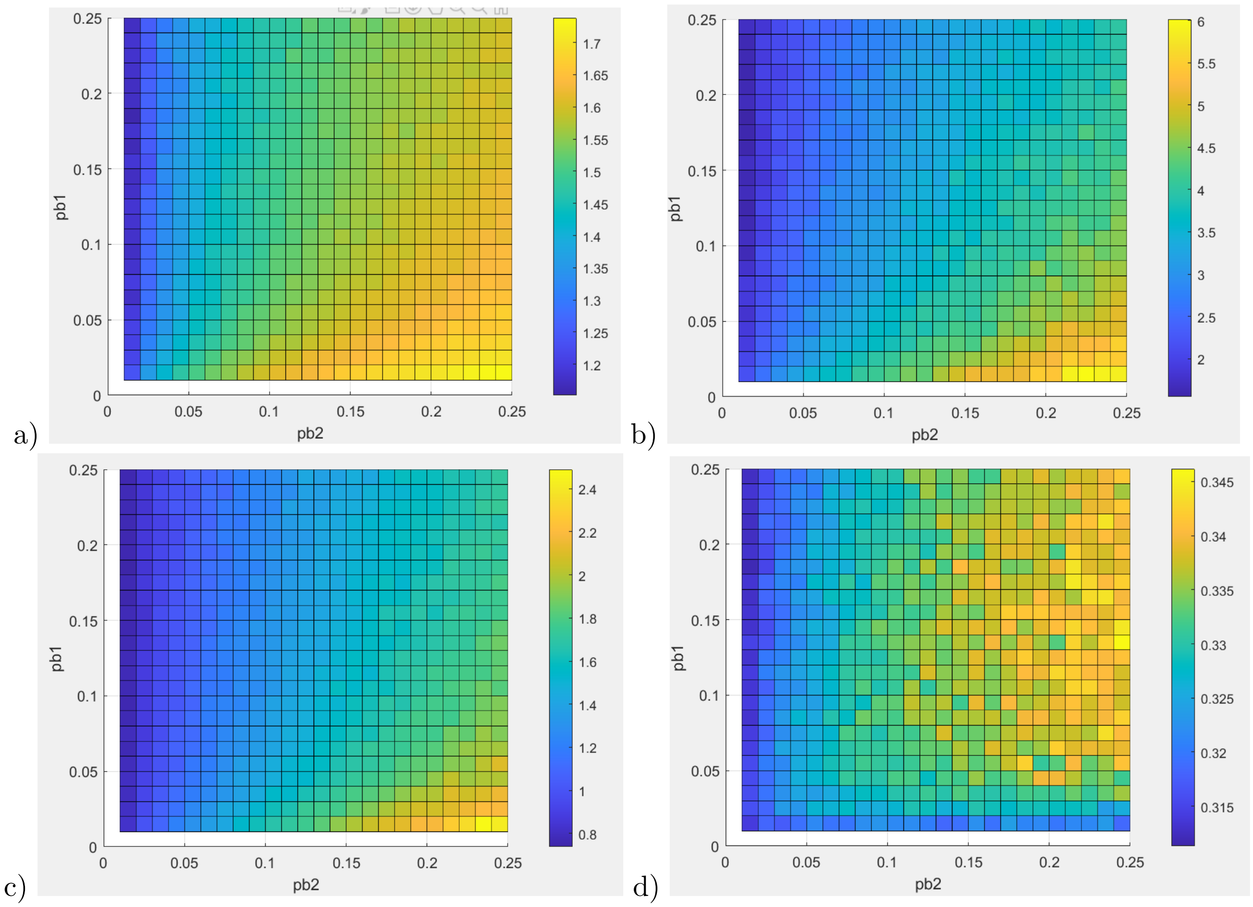

Figure S1: Effect of the ratio of probability $pb1$ values against $pb2$ values on bacterial attachement characteristics using 2D one species model for a) roughness coefficient, b) maximum thickness, c) mean thickness on bacteria only, d) mean thickness on whole surface (including voids). 50 simulations were performed with $Nb_{cell} = 100$ and $ps = 0.005$. Color grade indicates the average value of each bacterial attachement criterium according to pb or ps values.

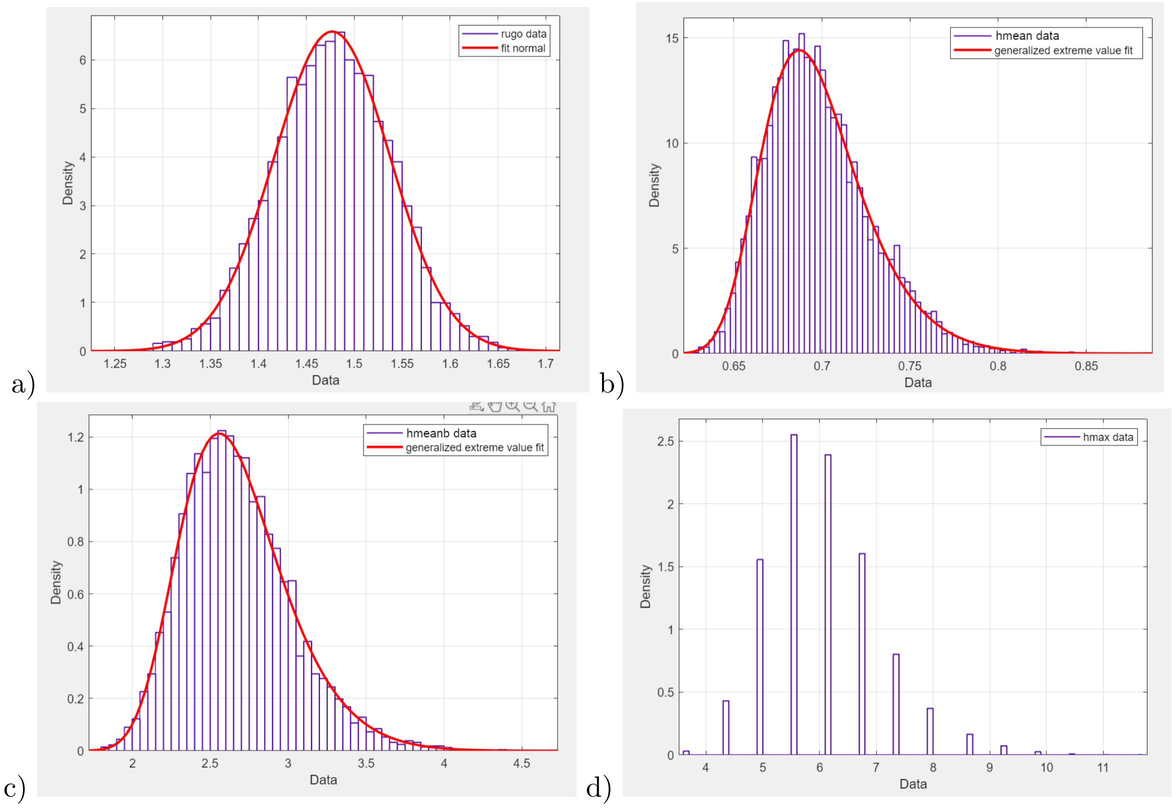

Figure S2: Distribution of the values with $N = 10000$ simulations, $Nb_{cell} = 200$, $ps = 0.001$, $pb1 = pb2 = 0.1$ for a) the rugosity coefficient of normal law $N(1.48, 0.06)$, b) the mean thickness of generalized extreme law ($\mu = 0.6855$, $\sigma = 0.026$, $\zeta = -0.077$), c) the mean thickness on biofilm of generalized extreme law ($\mu = 2.525$, $\sigma = 0.305$, $\zeta = -0.098$), d) the maximum thickness of discrete law because its value is a multiple of dx.

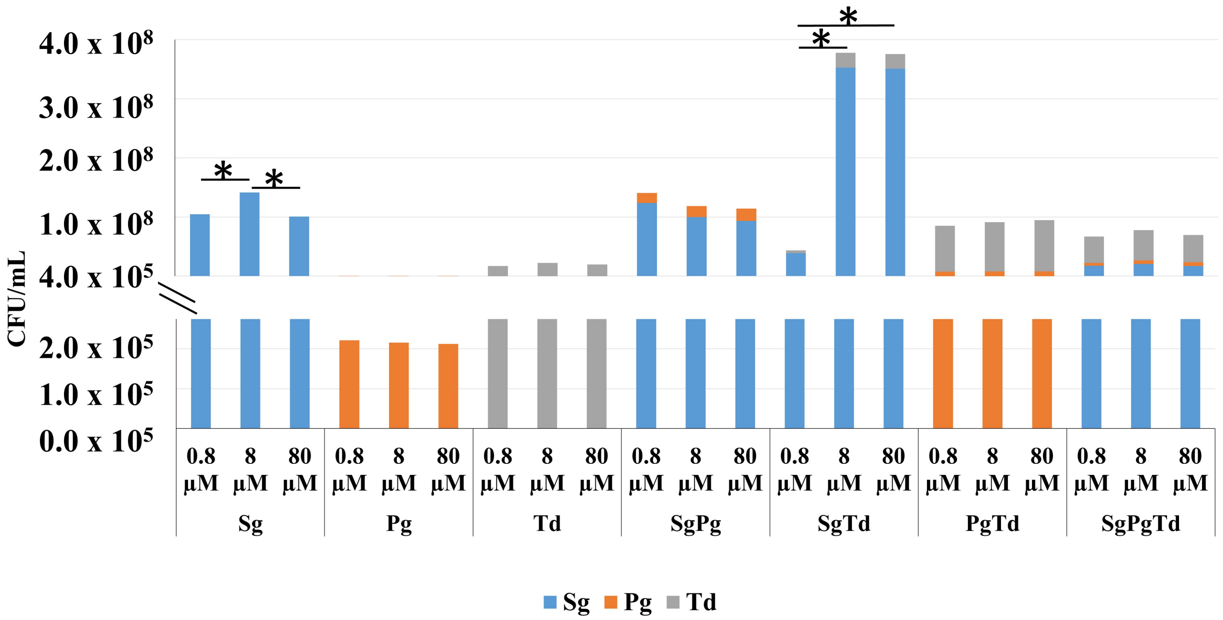

Figure S3: Comparison between mono-, dual- and three-species conditions. Initial attachment for each species were assessed for mono-species (*S. gordonii*-Sg, *P. gingivalis*- Pg and *T. denticola*-Td), dual-species (*S. gordonii*-*P. gingivalis*: SgPg, *S. gordonii*-*T. denticola*: SgTd, *P. gingivalis*-*T. denticola*: PgTd) and three-species (*S. gordonii*-*P. gingivalis*-*T. denticola*: SgPgTd) 2-hour sessile cells at sub-optimal (0.8 μM), optimal (8 μM) and excess iron (80 μM) levels. * indicates P-value < 0.05.

Iron [μM]	Bacterium	Mono-species attachment	Dual-species attachment			Three-species attachment
			S. gordonii *P. gingivalis*	*S. gordonii* *T. denticola*	*P. gingivalis* *T. denticola*	
0.8	*S. gordonii*	1.05E+08 ± 2.82E+07	1.16E+08 ± 1.02E+08	4.20E+07 ± 1.33E+07		1.79E+07 ± 2.44E+06
	P. gingivalis	2.21E+05 ± 9.76E+04	1.47E+07 ± 1.46E+07		7.66E+06 ± 3.99E+06	4.77E+06 ± 7.36E+05
	T. denticola	1.74E+07 ± 4.36E+06		5.02E+06 ± 2.68E+06	8.07E+07 ± 3.43E+07	4.46E+07 ± 1.45E+07
8	*S. gordonii*	1.42E+08 ± 4.11E+07	8.76E+07 ± 7.32E+07	3.53E+08 ± 7.21E+07		2.07E+07 ± 9.75E+06
	P. gingivalis	2.16E+05 ± 6.79E+04	1.62E+07 ± 1.42E+07		7.33E+06 ± 3.36E+06	6.27E+06 ± 1.35E+06
	T. denticola	2.23E+07 ± 8.29E+06		2.55E+07 ± 1.43E+07	9.39E+07 ± 5.01E+07	5.10E+07 ± 1.06E+07
80	*S. gordonii*	1.01E+08 ± 2.20E+07	7.86E+07 ± 6.64E+07	3.72E+08 ± 1.05E+08		1.71E+07 ± 6.27E+06
	P. gingivalis	2.13E+05 ± 7.77E+04	1.83E+07 ± 1.43E+07		8.10E+06 ± 3.77E+06	6.76E+06 ± 8.74E+05
	T. denticola	2.00E+07 ± 6.65E+06		2.44E+07 ± 1.14E+07	9.15E+07 ± 5.02E+07	4.67E+07 ± 9.37E+06

Table S3: Concentration of each species (CFU/mL) in mono-species and multispecies biofilms at 0.8, 8 and 80 μM of iron.

Bacteria	Data	$biovol[\mu m^3/\mu m^2]$	$hmean[\mu m]$	$rough$	$hmeanb[\mu m]$	$hmax[\mu m]$
Sg	2D simulations	1.11	1.26±0.05	1.32±0.07	3.65±0.43	8.34±1.38
	3D simulations	1.20	1.27±0.00	1.25±0.04	3.19±0.17	9.34±0.77
	experiments	1.54±0.86	1.26±0.05	1.35±0.33	3.54±0.51	8.31±1.71
Pg	2D simulations	0.09	0.10±0.01	1.91±0.02	2.40±0.76	4.26±1.30
	3D simulations	0.09	0.10±0.00	1.92±0.01	2.37±0.30	6.41±0.89
	experiments	0.13±0.07	0.10±0.05	1.92±0.04	2.50±0.35	5.98±0.45
Td	2D simulations	0.12	0.15±0.02	1.94±0.02	5.10±1.71	9.59±2.94
	3D simulations	0.11	0.14±0.00	1.94±0.01	4.72±0.50	14.06±1.76
	experiments	0.12±0.05	0.14±0.06	1.94±0.03	5.17±1.69	13.28±4.64
SgPg	2D simulations	1.05	1.23±0.05	1.41±0.07	4.12±0.52	9.56±1.47
	3D simulations	1.09	1.20±0.01	1.41±0.03	3.90±0.19	12.61±0.90
	experiments	1.28±0.38	1.20±0.30	1.42±0.17	4.09±0.61	11.83±1.13
SgTd	2D simulations	2.46	2.78±0.08	0.98±0.09	5.01±0.53	11.03±1.38
	3D simulations	2.55	2.67±0.01	0.89±0.05	4.21±0.22	11.70±0.84
	experiments	3.11±0.53	2.66±0.44	0.92±0.13	4.17±0.28	11.52±1.22
PgTd	2D simulations	0.34	0.41±0.04	1.85±0.03	5.62±1.32	11.60±2.71
	3D simulations	0.37	0.42±0.01	1.84±0.02	5.39±0.54	16.61±1.61
	experiments	0.49±0.18	0.42±0.15	1.71±0.09	2.84±0.33	8.75±1.02
SgPgTd	2D simulations	1.72	1.91±0.06	1.30±0.11	5.45±0.95	11.75±2.08
	3D simulations	1.75	1.90±0.01	1.33±0.04	5.43±0.36	16.65±1.32
	experiments	1.77±0.43	1.86±0.46	1.32±0.10	5.14±0.58	15.74±0.96

Table S4: Comparison of 2D simulation (mean and standard deviation on $N = 100$ simulations), 3D simulation (mean and standard deviation on $N = 100$ simulations) and experimental results at 8 μM of iron.
